# A standard calculation methodology for human doubly labeled water studies

**DOI:** 10.1016/j.xcrm.2021.100203

**Published:** 2021-02-16

**Authors:** John R. Speakman, Yosuke Yamada, Hiroyuki Sagayama, Elena S.F. Berman, Philip N. Ainslie, Lene F. Andersen, Liam J. Anderson, Lenore Arab, Issaad Baddou, Kweku Bedu-Addo, Ellen E. Blaak, Stephane Blanc, Alberto G. Bonomi, Carlijn V.C. Bouten, Pascal Bovet, Maciej S. Buchowski, Nancy F. Butte, Stefan G.J.A. Camps, Graeme L. Close, Jamie A. Cooper, Seth A. Creasy, Sai Krupa Das, Richard Cooper, Lara R. Dugas, Cara B. Ebbeling, Ulf Ekelund, Sonja Entringer, Terrence Forrester, Barry W. Fudge, Annelies H. Goris, Michael Gurven, Catherine Hambly, Asmaa El Hamdouchi, Marije B. Hoos, Sumei Hu, Noorjehan Joonas, Annemiek M. Joosen, Peter Katzmarzyk, Kitty P. Kempen, Misaka Kimura, William E. Kraus, Robert F. Kushner, Estelle V. Lambert, William R. Leonard, Nader Lessan, David S. Ludwig, Corby K. Martin, Anine C. Medin, Erwin P. Meijer, James C. Morehen, James P. Morton, Marian L. Neuhouser, Theresa A. Nicklas, Robert M. Ojiambo, Kirsi H. Pietiläinen, Yannis P. Pitsiladis, Jacob Plange-Rhule, Guy Plasqui, Ross L. Prentice, Roberto A. Rabinovich, Susan B. Racette, David A. Raichlen, Eric Ravussin, Rebecca M. Reynolds, Susan B. Roberts, Albertine J. Schuit, Anders M. Sjödin, Eric Stice, Samuel S. Urlacher, Giulio Valenti, Ludo M. Van Etten, Edgar A. Van Mil, Jonathan C.K. Wells, George Wilson, Brian M. Wood, Jack Yanovski, Tsukasa Yoshida, Xueying Zhang, Alexia J. Murphy-Alford, Cornelia U. Loechl, Edward L. Melanson, Amy H. Luke, Herman Pontzer, Jennifer Rood, Dale A. Schoeller, Klaas R. Westerterp, William W. Wong, the IAEA DLW database group

**Affiliations:** 1 Center for Energy Metabolism and Reproduction, Shenzhen Institutes of Advanced Technology, Chinese Academy of Sciences, Shenzhen, China; 2 Institute of Biological and Environmental Sciences, University of Aberdeen, Aberdeen, UK; 3 State Key Laboratory of Molecular Developmental Biology, Institute of Genetics and Developmental Biology, Chinese Academy of Sciences, Beijing, China; 4 CAS Center of Excellence in Animal Evolution and Genetics, Kunming, China; 5 National Institute of Health and Nutrition, National Institutes of Biomedical Innovation, Health and Nutrition, Tokyo, Japan; 6 Institute for Active Health, Kyoto University of Advanced Science, Kyoto, Japan; 7 Faculty of Health and Sport Sciences, University of Tsukuba, Ibaraki, Japan; 8 Berman Scientific Consulting, Mountain View, CA, USA; 9 Research Institute for Sport and Exercise Sciences, Liverpool John Moores University, Liverpool, UK; 10 Department of Nutrition, Institute of Basic Medical Sciences, University of Oslo, 0317 Oslo, Norway; 11 Crewe Alexandra Football Club, Crewe, UK; 12 David Geffen School of Medicine, University of California, Los Angeles, Los Angeles, CA, USA; 13 Unité Mixte de Recherche en Nutrition et Alimentation, CNESTEN- Université Ibn Tofail URAC39, Regional Designated Center of Nutrition Associated with AFRA/IAEA, Rabat, Morocco; 14 Department of Physiology, Kwame Nkrumah University of Science and Technology, Kumasi, Ghana; 15 Maastricht University, Maastricht, the Netherlands; 16 Nutritional Sciences, University of Wisconsin, Madison, WI, USA; 17 Institut Pluridisciplinaire Hubert Curien, CNRS Université de Strasbourg, UMR7178, Strasbourg, France; 18 Phillips Research, Eindhoven, the Netherlands; 19 Department of Biomedical Engineering and Institute for Complex Molecular Systems, Eindhoven University of Technology, Eindhoven, the Netherlands; 20 Institute of Social and Preventive Medicine, Lausanne University Hospital, Lausanne, Switzerland; 21 Division of Gastroenterology, Hepatology and Nutrition, Department of Medicine, Vanderbilt University, Nashville, TN, USA; 22 Department of Pediatrics, Baylor College of Medicine, USDA/ARS Children’s Nutrition Research Center, Houston, TX, USA; 23 Division of Endocrinology, Metabolism and Diabetes, University of Colorado Anschulz Medical Campus, Aurora, CO, USA; 24 Friedman School of Nutrition Science and Policy, Tufts University, 150 Harrison Avenue, Boston, MA, USA; 25 Department of Public Health Sciences, Parkinson School of Health Sciences and Public Health, Loyola University, Maywood, IL, USA; 26 Boston Children’s Hospital, Boston, MA, USA; 27 Department of Sport Medicine, Norwegian School of Sport Sciences, Oslo, Norway; 28 Charité – Universitätsmedizin Berlin, corporate member of Freie Universität Berlin, Humboldt-Universität zu Berlin, and Berlin Institute of Health (BIH), Institute of Medical Psychology, Berlin, Germany; 29 University of California, Irvine, Irvine, CA, USA; 30 Solutions for Developing Countries, University of the West Indies, Mona, Kingston, Jamaica; 31 University of Glasgow, Glasgow, UK; 32 Department of Anthropology, University of California, Santa Barbara, Santa Barbara, CA, USA; 33 Central Health Laboratory, Ministry of Health and Wellness, Port Louis, Mauritius; 34 Pennington Biomedical Research Center, Baton Rouge, LA, USA; 35 Department of Medicine, Duke University, Durham, NC, USA; 36 Northwestern University, Chicago, IL, USA; 37 Research Unit for Exercise Science and Sports Medicine, University of Cape Town, Cape Town, South Africa; 38 Department of Anthropology, Northwestern University, Evanston, IL, USA; 39 Imperial College London Diabetes Centre, Imperial College London, London, UK; 40 Department of Nutrition and Public Health, Faculty of Health and Sport Sciences, University of Agder, 4630 Kristiansand, Norway; 41 The FA Group, Burton-Upon-Trent, Staffordshire, UK; 42 Division of Public Health Sciences, Fred Hutchinson Cancer Research Center and School of Public Health, University of Washington, Seattle, WA, USA; 43 Moi University, Eldoret, Kenya; 44 University of Global Health Equity, Kigali, Rwanda; 45 Helsinki University Central Hospital, Helsinki, Finland; 46 University of Brighton, Eastbourne, UK; 47 Department of Physiology, Kwame Nkrumah University of Science and Technology, Kumasi, Ghana; 48 Department of Nutrition and Movement Sciences, Maastricht University, Maastricht, the Netherlands; 49 University of Edinburgh, Edinburgh, UK; 50 Biological Sciences and Anthropology, University of Southern California, Los Angeles, CA, USA; 51 Centre for Cardiovascular Sciences, Queen’s Medical Research Institute, University of Edinburgh, Edinburgh, UK; 52 University of Tilburg, Tilburg, the Netherlands; 53 Department of Nutrition, Exercise and Sports, Copenhagen University, Copenhagen, Denmark; 54 Stanford University, Stanford, CA, USA; 55 Department of Anthropology, Baylor University, Waco, TX, USA; 56 Maastricht and Lifestyle Medicine Center for Children, Jeroen Bosch Hospital’s-Hertogenbosch, Maastricht University, Maastricht, the Netherlands; 57 Population, Policy and Practice Research and Teaching Department, UCL Great Ormond Street Institute of Child Health, London, UK; 58 University of California, Los Angeles, Los Angeles, CA, USA; 59 Department of Human Behavior, Ecology, and Culture, Max Planck Institute for Evolutionary Anthropology, Leipzig, Germany; 60 Growth and Obesity, Division of Intramural Research, NIH, Bethesda, MD, USA; 61 Nutritional and Health Related Environmental Studies Section, Division of Human Health, International Atomic Energy Agency, Vienna, Austria; 62 Eastern Colorado VA Geriatric Research, Education and Clinical Center, Aurora, CO, USA; 63 Division of Geriatrics, University of Colorado Anschutz Medical Campus, Aurora, CO, USA; 64 Division of Epidemiology, Department of Public Health Sciences, Loyola University School of Medicine, Maywood, IL, USA; 65 Evolutionary Anthropology, Duke University, Durham, NC, USA; 66 Duke Global Health Institute, Duke University, Durham, NC, USA; 67 Biotech Center and Nutritional Sciences, University of Wisconsin, Madison, WI, USA; 68 School of Nutrition and Translational Research in Metabolism, University of Maastricht, Maastricht, the Netherlands

**Keywords:** total energy expenditure, free-living, validation, doubly labeled water

## Abstract

The doubly labeled water (DLW) method measures total energy expenditure (TEE) in free-living subjects. Several equations are used to convert isotopic data into TEE. Using the International Atomic Energy Agency (IAEA) DLW database (5,756 measurements of adults and children), we show considerable variability is introduced by different equations. The estimated rCO_2_ is sensitive to the dilution space ratio (DSR) of the two isotopes. Based on performance in validation studies, we propose a new equation based on a new estimate of the mean DSR. The DSR is lower at low body masses (<10 kg). Using data for 1,021 babies and infants, we show that the DSR varies non-linearly with body mass between 0 and 10 kg. Using this relationship to predict DSR from weight provides an equation for rCO_2_ over this size range that agrees well with indirect calorimetry (average difference 0.64%; SD = 12.2%). We propose adoption of these equations in future studies.

## Highlights

5,756 doubly labeled water (DLW) measures highlight variation from calculation equation
We derive here new equations for calculating CO_2_ production when using DLW
These equations outperform previous equations in validation studies
We recommend these equations should be adopted in future studies using DLW in humans

## Graphical Abstract

**Figure:**
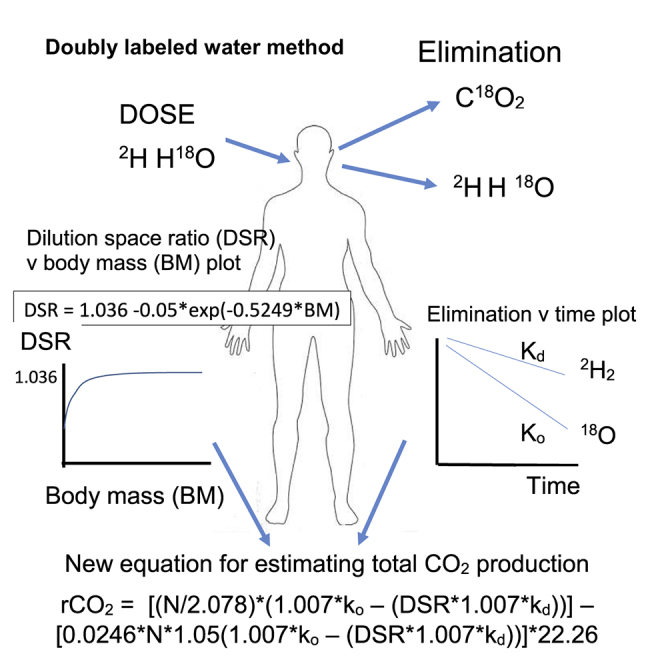

## Teaser

Speakman et al. use a large database of doubly labeled water measurements to show the choice of equation for the calculation of energy expenditure introduces significant variation into the final estimate. They then derive new equations that outperform previous equations in validation studies against chamber calorimetry.

## Introduction

The doubly labeled water (DLW) method^1^^,^^2^ is an isotope-based technique for measuring rCO_2_ in free-living animals and humans.^3^ The method is based on the observation that the oxygen in respiratory CO_2_ is in complete isotopic equilibrium with the oxygen in body water. Hence, isotopically labeled oxygen introduced into the body water is eliminated as both water and CO_2_. In contrast, a simultaneously introduced label of hydrogen (such as deuterium) will be predominantly eliminated as water. The difference in elimination rates of the two isotopes (hence “doubly labeled” water) gives a measure of rCO_2_. If the respiratory quotient (RQ) (the ratio of CO_2_ production to O_2_ consumption) or food quotient (FQ) (the proportions of fat, protein, and carbohydrate in the diet) is known, the rCO_2_ can be converted to estimated energy expenditure using standard equations.

The prohibitive cost of the isotopes limited early use of the method to small animals.^4^ Advances in mass spectrometry, which reduced the required dose, along with the declining cost of the isotopes enabled the first applications to humans in the early 1980s.^5^, ^6^, ^7^ Since then, use of the method has grown steadily with currently approximately 100 papers published using the method annually.^8^ However, costs continue to keep sample sizes in most studies relatively small (typically less than 50 individuals). There has been an impetus in the last few years, therefore, to combine data across studies to extend or modify conclusions about the main factors driving energy demands.^9^^,^^10^

The simple description of the technique above belies a great deal of complexity in its theoretical basis.^2^^,^^3^^,^^10^^,^^11^ For example, isotopes fractionate as they leave the body, so that lighter isotopes are preferentially lost. This effect needs to be accounted for in the calculation. Another issue is that the isotopes are assumed to be turning over in the body water pool. The body water pool can be measured from the dilution space of the isotopic doses, but the dilution space of 18oxygen (N_O_) differs from that of deuterium (N_d_), and both differ slightly from the total body water (TBW). The oxygen dilution space is about 1% larger than the TBW although the hydrogen dilution space is about 4% larger. This difference stems primarily from hydrogen in body water exchanging with labile hydrogen in proteins and other organic molecules in the body. The relationship between N_d_, N_o_, and TBW affect the calculation of rCO_2_, and thus, the dilution space ratio (DSR), which is equal to N_d_/ N_O_, turns out to be a critical parameter in DLW studies.

A final complexity that must be considered is the choice of equation used to calculate rCO_2_. Although there are only four basic parameters that are derived from the isotope elimination measurements (the two elimination constants for 18oxygen [k_O_] and deuterium [k_d_] and the two isotope dilution spaces [N_O_ and N_d_]), the best approach combining these parameters to estimate rCO_2_ was a matter of considerable debate throughout the late 1980s and 1990s.^3^ These discussions never reached a broad consensus, and hence, different studies have subsequently combined the parameters in slightly different ways. Such differences are largely irrelevant if the objective is to compare groups within a single study. However, if absolute values of energy demand are required, such as might be needed if the DLW method is being utilized as a validation method (for example, for measurements of habitual food intake), to compare total energy expenditure (TEE) across cultures and lifestyles, or if comparisons are made to previous studies, the differences in calculation could be significant. The consequences of this variability have never been thoroughly evaluated but have been assumed to be small relative to the biological variation under study. In this paper, we evaluate the impact of using different equations and derive new standard equations based on performance in validation studies for use in future studies. We address this issue first for studies of children, adolescents, and adults and then for studies of small infants and babies.

## Results and discussion

### Children, adolescents, and adults

We have compiled in the International Atomic Energy Agency (IAEA) DLW database (v3.1; https://www.dlwdatabase.org) individual data from 119 DLW studies comprising a total of 6,246 measurements of individuals aged 2–96 years.^8^ For 5,756 of these measurements, we have access to the individual values of k_o_, k_d_, N_o_, and N_d_, allowing us to recalculate rCO_2_ using a single equation, and compare these to the original estimates made using a diversity of calculation methods. To choose the best equation for the common calculation, we compiled data from six validation studies involving 61 adult humans, where rCO_2_ by DLW has been compared with simultaneous indirect calorimetry (Table 1).^12^, ^13^, ^14^, ^15^, ^16^, ^17^ This comparison yielded three equations where rCO_2_ did not differ significantly from the chamber values (Table 1).^3^^,^^18^, ^19^, ^20^, ^21^, ^22^ The equation with the lowest average deviation was derived from an analysis of dilution space ratios in Sagayama et al.^20^ Using the average dilution space ratio of 1.036, we modified the original Equation A6 proposed by Schoeller et al.^15^ and derived a new equation here, for which the average discrepancy between the DLW estimates of rCO_2_ and simultaneous chamber estimates was −0.4% (SD = 7.6%; Table 1).

**Table 1:** Validation results for carbon dioxide production (rCO_2_) for 61 individuals measured using the doubly labeled water method simultaneous to chamber calorimetry

Source	ID	BM	VCO2 IC	Schoeller 1988		Racette et al., 1994		Sagayama et al., 2016		Speakman 1997		Speakman et al., 1993		Coward and Prentice 1985	
		kg	L/d	L/d	%	L/d	%	L/d	%	L/d	%	L/d	%	L/d	%
Schoeller and Webb^12^	M17	67.5	566.7	579.3	2.2	571.4	0.8	567.5	0.1			548.9	−3.2		
	F25	72.0	439.0	448.9	2.2	440.4	0.3	436.2	−0.6			417.9	−4.8		
	F27	57.1	436.8	382.1	−12.5	374.2	−14.3	370.3	−15.2			353.6	−19.0		
	M28	67.5	611.5	608.2	−0.5	596.6	−2.4	590.9	−3.4			565.8	−7.5		
	M58	88.2	486.1	521.8	7.4	514.3	5.8	510.5	5.0			493.0	1.4		
Westerterp et al.^13^	1	73.2	508.0	495.0	−2.6	487.1	−4.1	483.2	−4.9			465.4	−8.4		
	2	77.9	479.0	506.5	5.7	498.2	4.0	494.2	3.2			475.8	−0.7		
	3	57.6	356.0	352.0	−1.1	346.5	−2.7	343.8	−3.4			331.4	−6.9		
	4	72.0	457.0	441.4	−3.4	435.5	−4.7	432.6	−5.3			418.6	−8.4		
	5	58.1	437.0	422.9	−3.2	414.3	−5.2	410.1	−6.2			391.8	−10.3		
	6A	75.6	894.0	919.0	2.8	907.5	1.5	901.9	0.9			874.1	−2.2		
	7A	64.7	818.0	931.9	13.9	920.6	12.5	915.1	11.9			887.7	8.5		
	8A	71.0	981.0	947.5	−3.4	934.2	−4.8	927.7	−5.4			896.6	−8.6		
	9A	77.9	1,104.0	1,085.9	−1.6	1,070.4	−3.0	1,062.8	−3.7			1,026.8	−7.0		
Seale et al.^14^	1	100.4	531.0	550.7	3.7	538.0	1.3	531.7	0.1			505.3	−4.8		
	2	50.3	392.0	407.4	3.9	398.5	1.7	394.2	0.6			375.5	−4.2		
	3	59.0	331.0	343.2	3.7	336.3	1.6	333.0	0.6			318.4	−3.8		
	4	52.6	451.0	442.1	−2.0	427.3	−5.3	420.1	−6.9			391.1	−13.3		
	5	82.7	530.0	545.9	3.0	535.0	0.9	529.7	−0.1			506.4	−4.5		
	6	86.2	550.0	545.2	−0.9	530.4	−3.6	523.1	−4.9			493.1	−10.3		
	7	87.4	515.0	531.0	3.1	522.2	1.4	517.9	0.6			498.2	−3.3		
	8	47.8	403.0	395.8	−1.8	383.8	−4.8	378.0	−6.2			354.2	−12.1		
	9	79.9	494.0	511.1	3.5	503.2	1.9	499.3	1.1			481.3	−2.6		
Schoeller et al.^15^	ID	75.3	559.0	570.6	2.1	564.5	1.0	561.1	0.4			543.4	−2.8		
	NM	75.6	614.0	598.5	−2.5	591.0	−3.7	587.3	−4.4			568.5	−7.4		
	ED	76.3	633.0	591.5	−6.6	582.8	−7.9	578.4	−8.6			557.9	−11.9		
	MK	69.5	541.0	543.6	0.5	531.5	−1.8	526.5	−2.7	537.2	−0.7	506.1	−6.5	529.3	−2.2
	JD	64.1	504.0	440.0	−12.7	432.7	−14.1	428.8	−14.9	438.3	−13.0	410.7	−18.5	340.5	−32.4
	DM	73.3	566.0	650.1	14.9	640.7	13.2	636.9	12.5	659.6	16.5	619.8	9.5	581.6	2.8
	AB	56.7	468.0	460.2	−1.7	452.6	−3.3	449.5	−4.0	463.3	−1.0	435.4	−7.0	443.6	−5.2
	LC	85.2	626.0	656.6	4.9	643.1	2.7	637.9	1.9	654.6	4.6	616.8	−1.5	632.3	1.0
	DP	63.1	529.0	519.6	−1.8	512.0	−3.2	508.7	−3.8	525.5	−0.7	493.4	−6.7	515.4	−2.6
Ravussin et al.^16^	1	124.6	499.0	462.1	−7.4	452.6	−9.3	448.6	−10.1	415.0	−16.8	432.0	−13.4	398.7	−20.1
	2	61.4	356.0	413.3	16.1	404.7	13.7	401.2	12.7	370.9	4.2	386.2	8.5	318.8	−10.5
	3	137.6	535.0	556.6	4.0	543.2	1.5	537.5	0.5	483.4	−9.7	514.2	−3.9	443.9	−17.0
	4	80.9	393.0	503.8	28.2	489.9	24.7	483.9	23.1	422.8	7.6	459.9	17.0	321.3	−18.2
	5	101.8	370.0	402.3	8.7	393.6	6.4	389.9	5.4	357.8	−3.3	374.8	1.3	318.5	−13.9
	6	139.9	424.0	427.7	0.9	420.1	−0.9	416.9	−1.7	393.7	−7.1	403.4	−4.9	384.7	−9.3
	7	190.9	711.0	733.7	3.2	718.0	1.0	711.4	0.0	653.8	−8.0	683.9	−3.8	541.5	−23.8
	8	95.8	480.0	590.9	23.1	575.0	19.8	568.0	18.3	498.8	3.9	540.4	12.6	396.9	−17.3
	9	151.5	672.0	683.3	1.7	662.0	−1.5	652.5	−2.9	551.9	−17.9	615.6	−8.4	510.6	−24.0
	10	68.6	373.0	406.3	8.9	390.4	4.7	383.1	2.7	300.2	−19.5	355.6	−4.7	277.5	−25.6
	11	69.4	332.0	354.2	6.7	344.4	3.7	340.0	2.4	296.4	−10.7	323.0	−2.7	234.7	−29.3
	12	80.1	403.0	468.0	16.1	457.6	13.5	453.2	12.5	413.8	2.7	435.1	8.0	361.5	−10.3
Melanson et al.^17^	1	63.0	310.6	299.4	−3.6	291.7	−6.1	286.6	−7.7	280.0	−9.8	263.8	−15.1	285.1	−8.2
	2	82.8	457.4	447.0	−2.3	440.4	−3.7	436.6	−4.6	445.0	−2.7	418.1	−8.6	420.4	−8.1
	3	74.8	455.8	476.2	4.5	467.5	2.6	463.5	1.7	474.2	4.0	445.5	−2.3	429.6	−5.7
	4	61.0	346.8	361.6	4.3	354.6	2.2	351.0	1.2	356.6	2.8	335.2	−3.4	324.2	−6.5
	5	93.8	471.3	465.4	−1.2	454.0	−3.7	449.0	−4.7	456.0	−3.2	428.7	−9.0	389.9	−17.3
	6	48.9	293.4	325.6	11.0	318.5	8.6	314.6	7.2	316.0	7.7	297.2	1.3	291.0	−0.8
	7	53.3	349.9	352.7	0.8	343.6	−1.8	339.1	−3.1	340.1	−2.8	320.0	−8.5	298.1	−14.8
	8	91.5	444.2	447.8	0.8	437.8	−1.4	433.7	−2.4	444.1	0.0	417.3	−6.1	385.0	−13.3
	9	71.6	442.8	429.6	−3.0	418.0	−5.6	412.6	−6.8	415.5	−6.2	390.9	−11.7	351.9	−20.5
	10	111.6	514.4	550.8	7.1	539.5	4.9	533.7	3.7	540.7	5.1	508.3	−1.2	489.8	−4.8
	11	95.0	437.1	540.4	23.6	526.7	20.5	519.7	18.9	520.7	19.1	489.9	12.1	461.6	5.6
	12	115.0	423.1	470.5	11.2	461.6	9.1	457.5	8.1	468.0	10.6	439.7	3.9	421.2	−0.5
	13	101.4	433.7	433.1	−0.1	423.7	−2.3	419.3	−3.3	426.5	−1.7	400.9	−7.6	376.7	−13.2
	14	73.9	473.4	443.0	−6.4	428.9	−9.4	422.8	−10.7	424.5	−10.3	399.5	−15.6	335.1	−29.2
	15	72.0	394.0	353.6	−10.3	344.8	−12.5	340.8	−13.5	344.8	−12.5	324.2	−17.7	296.4	−24.8
	16	61.7	353.8	345.7	−2.3	335.6	−5.2	331.0	−6.5	332.3	−6.1	312.6	−11.6	274.7	−22.4
	17	69.6	387.9	402.2	3.7	393.9	1.5	389.9	0.5	396.5	2.2	372.6	−3.9	354.4	−8.6
All subjects	N	61	61	61	61	61	61	61	61	35	35	61	61	35	35
	mean	80.5	497.52	509.71	2.74	499.49	0.60	494.69	−0.40	440.52	−2.08	473.96	−4.72	392.47	−12.89
	SD	26.2	152.69	155.18	7.97	153.74	7.74	153.11	7.67	98.67	9.05	149.72	7.51	95.32	9.94
	T				2.69		0.61		−0.4		1.36		−4.9		−7.7
	P				0.009		0.55		0.68		0.18		<0.001		<0.001

Source is the reference where the original validation data were published. ID is the ID from the original study. BM is the mean body mass of the individual in kg. rCO_2_ IC is the indirect calorimetry estimate of CO_2_ production in liters per day. For each DLW equation, the original data were used to calculate rCO_2_ and the % difference between these estimates and the chamber CO_2_ production is calculated. At the bottom of the table, the summary statistics across all 61 individuals are shown. Schoeller 1988 refers to Equation A6 in Schoeller et al.^15^ as modified in Schoeller.^18^ Racette et al., 1994 refers to Equation A6 in Schoeller et al.^15^ with the revised dilution space constant provided by Racette et al.^19^ Sagayama et al., 2016 refers to Equation A6 in Schoeller et al.^15^ with the revised dilution space constant provided by Sagayama et al.^20^ and detailed here as Equation 1. Speakman 1997 refers to Equation 17.41 in Speakman.^3^ Speakman et al., 1993 refers to Equation 3 in Speakman et al.,^21^ and Coward and Prentice 1985 refers to the two-pool equation in Coward and Prentice.^22^ For some of the studies, N_d_ was not available from the original validations. Because the equations by Speakman 1997 and Coward 1985 require individual estimates of N_d_, a comparison was not possible for these subjects, and the total statistics are based on n = 35. The t and p values refer to the difference of the mean difference from an expectation of 0 (single sample t test). Three equations produced estimates that were not significantly different to the chamber calorimetry data.

The new equation is as follows:

(Equation 1) $${\text{rCO}}_{2}=\left[\left(\text{N/}2.078\right)\text{∗}\left(1.007\text{∗}{\text{k}}_{\text{o}}\text{– }1.043\text{∗}{\text{k}}_{\text{d}}\right)\text{ – }\left(0.0246\text{∗N∗}1.05\left(1.007\text{∗}{\text{k}}_{\text{o}}\text{– }1.043\text{∗}{\text{k}}_{\text{d}}\right)\right)\right]\text{∗}22.26\text{,}$$

where

(Equation 2) $$\text{N }=\left[\left({\text{N}}_{\text{o}}\text{/}1.007\right)\;+\;\left({\text{N}}_{\text{d}}\text{/}1.043\right)\right]\text{/}2.$$

N is total body water. Using the dilution spaces of both isotopes to estimate N reduces the error due to analytical variation in the derivation of either isotope space alone. However, if it is felt that the analytical variation stems mostly from evaluation of the deuterium dilution space N_d_, then it is also acceptable to calculate N from the oxygen dilution space alone (N = N_o_ /1.007). The value 22.26 in Equation 1 is the gas constant for carbon dioxide. Note that this differs from the value used previously in all DLW equations for calculation of rCO_2_ of 22.4, which is erroneously high (by 0.7%) because CO_2_ does not show ideal gas behavior.^23^

Equation 1 can be simplified for calculation purposes to

(Equation 3) $${\text{rCO}}_{2}=0.4554\text{∗N∗}\left[\left(1.007\text{∗}{\text{k}}_{\text{o}}\right)-\left(1.043\text{∗}{\text{k}}_{\text{d}}\right)\right]\text{∗}22.26$$

or

(Equation 4) $${\text{rCO}}_{2}=\left[\text{N∗}\left(\left(0.45859\text{∗}{\text{k}}_{\text{o}}\right)-\left(0.47498\text{∗}{\text{k}}_{\text{d}}\right)\right)\right]\text{∗}22.26\text{,}$$

where k_o_ and k_d_ are in units of d^−1^, N_o_ and N_d_ are in mols, and rCO_2_ is in L/d.

We used the original RQ estimates from the publications to convert rCO_2_ to TEE using the Weir equation.^24^

(Equation 5) $$\text{TEE }\left(\text{MJ/d}\right){=\text{ rCO}}_{2}\text{∗}\left(1.106+\left(3.94\text{/RQ}\right)\right)\text{ ∗ }\left({4.184\text{/}10}^{3}\right).$$

**Figure 1:**
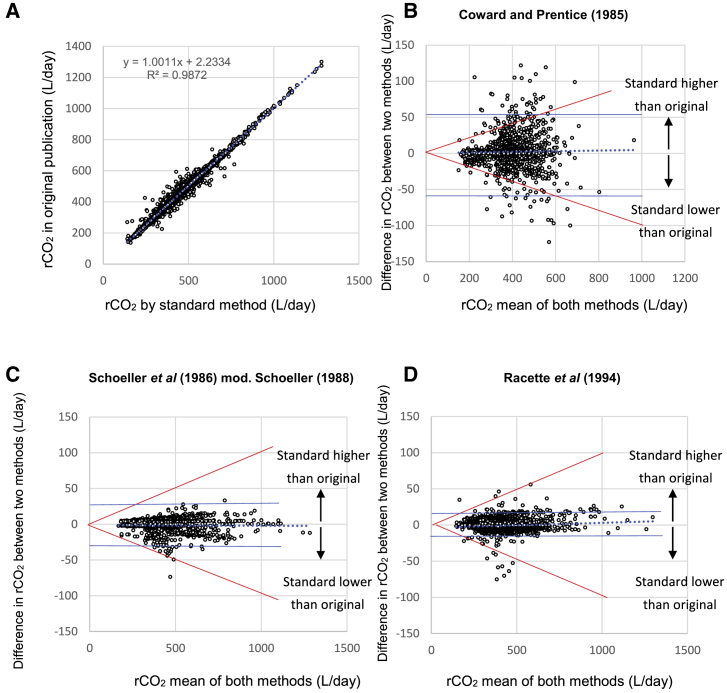
Comparison of published CO_2_ production by doubly labeled water to that by standard method (A) Relationship between CO_2_ production (L/d) for 5,756 individuals extracted from the original studies and the recalculated estimates using Equation 1. (B–D) Bland-Altman plots^25^ comparing the published rCO_2_ for studies using (B) the Coward and Prentice^22^ equation, (C) the Schoeller et al.^15^ A6 equation, and (D) the Racette et al.^19^ compared with the standard Equation 1 derived from Sagayama et al.^20^ In all plots, dotted line is average difference, and solid blue lines are plus and minus 2 SDs. The red lines define the boundary for plus and minus 10% difference between methods. Data refer to 5,756 adult individuals uploaded into the IAEA DLW database (v3.1).

Figure 1A shows the estimates of rCO_2_ from the original publications, plotted against estimates using Equation 1. Although there is a strong association between the estimates (r^2^ = 0.987), they do not yield identical rCO_2_ values. Because the equation based on Sagayama et al.^20^ was derived here, none of the studies in the database used this equation. Of the 5,756 individual data, the rCO_2_ of 1,024 (17.7%) was made using the equation of Coward and Prentice,^22^ 883 (15.3%) were made using the Schoeller et al.^15^ Equation A6 as modified in 1988,^18^ 3,770 (65.3%) were made using the Racette et al.^19^ equation, and 77 (1.3%) did not state the equation they used. The Racette et al.^19^ equation produces estimates very similar to those derived from Equation 1 (Table 1), and the discrepancy in the sample of 3,770 using this equation averaged 1.1% (SD 1.2). On average, the discrepancy when using the Schoeller et al.^15^ A6 equation was 1.8% (SD 1.6), and for the studies using the Coward and Prentice^22^ equation, it was 4.4% (SD 4.6).

We compared the rCO_2_ values calculated using the three main equations compared to Equation 1 using Bland-Altman plots (Figures 1B–1D).^25^ For all three equations, there was no systematic bias. However, the Coward and Prentice^22^ equation generated far more variable estimates than the other two equations. This is expected because that calculation utilizes individual values for N_o_ and N_d_ instead of using an average N_d_/N_o_ ratio, which is used in the other two equations and Equation 1. Indeed, of the 1,024 estimates using the Coward equation, 103 (10.0%) differed by more than 10% from the standard, compared to 1/883 (0.1%) for the Schoeller et al.^15^ equation and 12/3,770 (0.3%) for the Racette^19^ equation.

**Figure 2:**
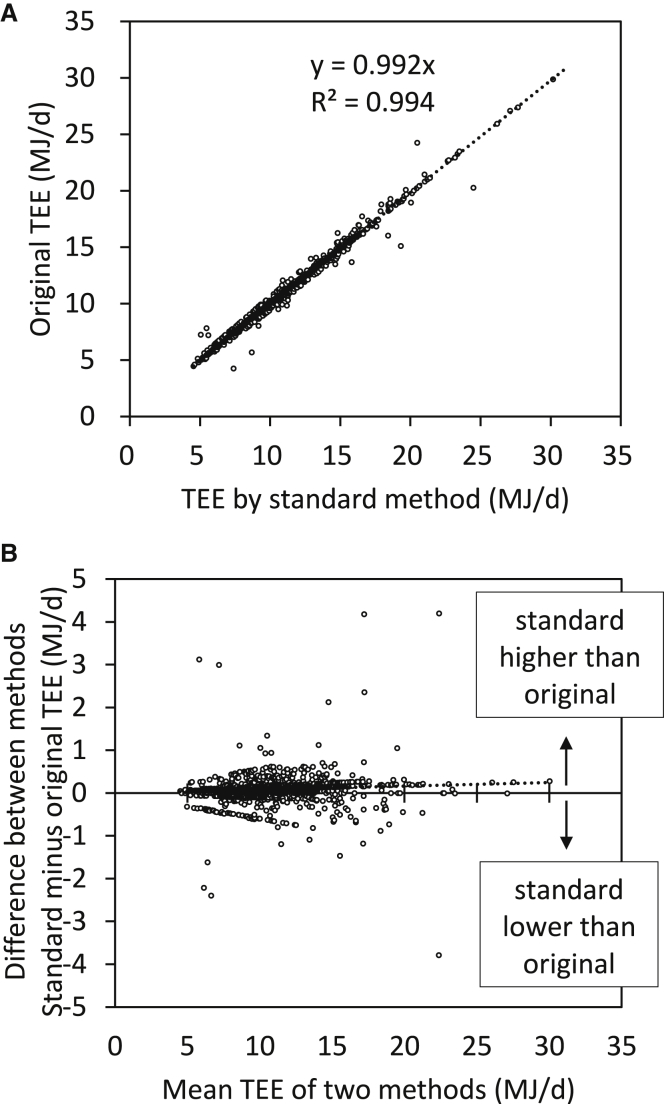
Comparison of published energy expenditure by DLW to that calculated by standard method (A) Relationship between the TEE (MJ/d) for 4,571 individual adults extracted from the original studies and the recalculated TEE using the Weir equation. (B) Bland-Altman plot^25^ comparing the published TEE with those generated using the recommended equation. Dotted line is average difference. Data refer to data for 4,571 adult individuals uploaded into the IAEA DLW database (v3.1). The sample size is lower than in Figure 1, because for some individuals, estimates of RQ or FQ were not available.

A second source of variation can be introduced by using alternative equations to convert rCO_2_ to TEE. This variation occurs even when the RQ is known. To evaluate the variation introduced from this source, we took the original rCO_2_ and converted this to TEE using the Weir equation. We then compared the recalculated TEE with the published values. The relationship between the recalculated and original TEE values (Figure 2A) was very good (r^2^ = 0.99), and the average discrepancy between estimates was only 0.08 MJ/d (SD = 0.19) or 0.8% (SD = 0.19). The absolute discrepancy excluding the sign of the difference was 0.11 MJ (1.1%; SD = 0.17). There was no significant trend in the discrepancy with the magnitude of the TEE (Figure 2B). When RQ is not known, the routine procedure is to approximate the RQ using the FQ. The errors involved in this approximation are beyond the scope of this paper and are not addressed here.

These data show that selection of the calculation method can introduce substantial variation into the individual and to a lesser extent average estimates of rCO_2_, as well as to variation in conversion of rCO_2_ to TEE. For comparisons made within studies, this discrepancy is unimportant. However, it may introduce problems when comparisons are attempted between studies or when the DLW method is used to validate other techniques, particularly when small sample sizes are employed. With some equations in common use, more than 10% of estimates are greater than 10% divergent from the equation that performs best in validation studies. Such differences between calculation methods across studies might be erroneously attributed to biological factors. This potential problem is compounded by the fact that some studies do not indicate the exact calculation methods they employed to derive rCO_2_ and TEE estimates. To overcome these issues, we recommend adoption of Equation 1 in future studies of children, adolescents, and adults to derive rCO_2_ and use of Equation 5 to convert this to TEE.

### Small infants and babies

**Figure 3:**
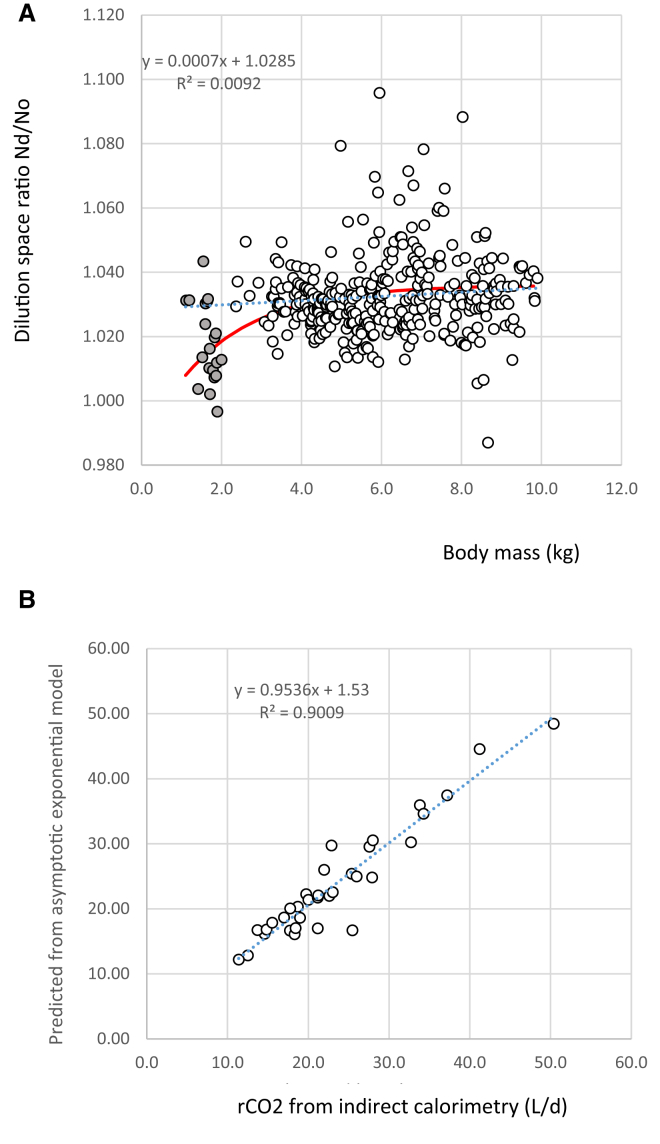
Dilution space ratio as a function of body mass and performance of new equation against indirect calorimetry (A) Dilution space ratios (the hydrogen dilution space N_d_ divided by the oxygen dilution space N_o_) of 332 babies weighing <10 kg from the IAEA DLW database v 3.1 (open circles) combined with data from validation studies in preterm and full-term babies (gray circles). For the sample from the database, there was a linear relationship (blue dotted line that marginally failed to reach significance p = 0.08). We fitted an asymptotic exponential to the combined dataset (red line; r^2^ = 6.4%; p < 0.03). (B) The results of validation studies of the DLW method in babies comparing the DLW estimates of CO_2_ production (rCO_2_) derived from a combination of Equations 9 and 10 presented here and rCO_2_ measured by indirect calorimetry. There was a strong linear relationship fitted by least-squares regression—dotted blue line, with r^2^ = 0.90.

The recommendation above refers to subjects aged ≥2 years. We have shown that the choice of equation has a significant impact on the resultant calculation of rCO_2_ and TEE and that the major factor driving this variation is the relative dilutions spaces of N_o_ and N_d_ (the dilution space ratio DSR = N_d_/N_o_; Figure 3). There is evidence that, at younger ages, the DSR is below the observed average of 1.036 in individuals aged >2 years.^20^^,^^26^ In a review of 36 studies of 1,131 young children, the weighted dilution space ratio averaged 1.031,^20^ which means that application of Equation 1 to younger individuals may yield underestimates of rCO_2_ and TEE.

There is a problem, however, in choosing the best equation to use in young children, and that is the limitation on performing validation experiments in this age group against gas exchange measurements by indirect calorimetry (chamber respirometry). Validation studies of DLW against indirect calorimetry will probably never be performed in young children because it would require the child to be isolated within a respirometry chamber for a protracted period lasting up to a week.

Nevertheless, a number of validation studies have been performed in preterm babies and small neonates (<2 kg), comparing continuous gas exchange with DLW.^27^, ^28^, ^29^ The problem, however, is that such very small children weighing less than 2 kg have an even lower DSR,^30^ averaging around 1.019, significantly lower than in infants weighing >2 kg.^26^^,^^31^ Hence, an equation based on this DSR might work well for small babies weighing less than 2 kg, but it might be unsuitable for infants weighing 2–10 kg. Fortunately, there is a single validation study of babies weighing 2–4.2 kg,^32^ which can assist in selection of the best equation in this size range.

We compiled data from the four available validation studies in babies and used the published data in these studies on isotope elimination rates of 18oxygen (k_o_) and deuterium (k_d_) and the respective dilution spaces (N_o_ and N_d_) to recalculate the rCO_2_ using five different alternative equations. We then derived two new equations in which we replaced the DSR in Equation 1 with either the value 1.019 or the value 1.031. These are, respectively, when the DSR = 1.019,

(Equation 6) $${\text{rCO}}_{2}=\left[\left(\text{N/}2.078\right)\text{∗}\left(1.007\text{∗}{\text{k}}_{\text{o}}\text{– }1.026\text{∗}{\text{k}}_{\text{d}}\right)\text{ – }\left(0.0246\text{∗N∗}1.05\left(1.007\text{∗}{\text{k}}_{\text{o}}\text{– }1.026\text{∗}{\text{k}}_{\text{d}}\right)\right)\right]\text{∗}22.26\text{,}$$

and when the DSR = 1.031,

(Equation 7) $${\text{rCO}}_{2}=\left[\left(\text{N/}2.078\right)\text{∗}\left(1.007\text{∗}{\text{k}}_{\text{o}}\text{– }1.038\text{∗}{\text{k}}_{\text{d}}\right)\text{ – }\left(0.0246\text{∗N∗}1.05\left(1.007\text{∗}{\text{k}}_{\text{o}}\text{– }1.038\text{∗}{\text{k}}_{\text{d}}\right)\right)\right]\text{∗}22.26.$$

In all the above cases, we used

(Equation 8) $$\text{N}{=\text{ N}}_{\text{o}}\text{/}1.007.$$

Although there have been relatively few validation studies of humans weighing less than 4 kg, there have been a large number of validation studies in small mammals and birds in this weight range (reviewed in Speakman^3^). Although such animals have dilution space ratios that do not differ from adult humans (around 1.036), the best equation in validation studies of such animals turns out to be based on a DSR of 1.0. This is because these animals have a significant efflux of deuterium in addition to water turnover that offsets the impact of the slightly different DSRs.^33^ Because this might also pertain in babies, we added into the evaluation the most widespread equation in use for small mammals and birds, which is Equation 7.17 from Speakman.^3^ Finally, we also added into the evaluation the equation of Coward and Prentice,^22^ which uses individual dilution spaces rather than a population average in the calculation.

Table 1 shows the results of the different equations when compared to indirect calorimetry for preterm infants (≤2 kg) and infants weighing >2 kg. The data show that, in the size range 0–2 kg, the best equation was based on the dilution space ratio 1.019 (Equation 6 above). The average difference between the rCO_2_ by indirect calorimetry and DLW using this equation was 0.5%. This was much better than the equation derived for children and adults (Equation 1), which gave an estimate 13.5% too low, and Equation 7 above, which gave an estimate 8.4% too low. The equation that performs best in validation studies of small mammals gave an estimate 10.1% too high, clearly indicating the physiological basis for this equation, although appropriate for birds and small non-human mammals, does not apply to neonatal humans and young infants.

In the size range 2–4 kg, the best equation was that based on the DSR of 1.031 (Equation 7). Equation 1 gave an estimate 8.5% too low. Equation 6 gave an estimate 6.5% too high, although the small animal equation gave an estimate 16.8% too high. These validation data therefore suggest that adoption of three different equations over different size ranges corresponding to different DSRs might be a possible solution to the issue of how to measure rCO_2_ by DLW. For individuals weighing <2 kg, the suggested equation would be Equation 6; for individuals weighing 2–10 kg, it would be Equation 7, and for individuals weighing >10 kg, it would be Equation 1.

This approach, however, is not very satisfactory, because it leads to confusion at the boundaries of the weight ranges. For example, for a 2-kg child, rCO_2_ calculated using Equation 6 differs from that calculated by Equation 7 by about 10%. To further explore the choice of DSR in the size range 0–10 kg, we extracted data from the IAEA DLW database^8^ for individuals in this size range. In fact, none of the individuals in the database weighed less than 2 kg, but there were 336 records of children weighing between 2.4 and 10 kg. The DSR for these individuals is plotted against the body weight in Figure 1A. The average DSR in this interval was 1.032 (SD = 0.0122), consistent with the previous suggestion of 1.031 (Sagayama et al.^20^). This DSR was significantly lower than the ratio established for heavier individuals of 1.036 (t = −5.72; p < 0.0001) and significantly higher than the ratio of 1.019 for preterm babies and neonates^30^ weighing less than 2 kg (t = 22.26; p < 0.001). There was a trend for a positive association between weight and DSR through the size range (regression r^2^ = 0.9%; p = 0.08). When we combined these data with those from the validation studies,^27^, ^28^, ^29^^,^^32^ there was a significant non-linear relationship between body mass (BM) (kg) and DSR. We fitted an asymptotic exponential model to these data constraining the asymptote to be 1.036 using a non-linear fitting function in the program MINITAB to estimate the unknown parameters. The resultant equation was

(Equation 9) DSR = 1.036 -0.05∗exp(-0.5249∗BM),

where BM is in kg.

A different approach then is to create an equation that combines this weight dependency with the standard equation, yielding

(Equation 10) $${\text{rCO}}_{2}=\left[\left(\text{N/}2.078\right)\text{∗}\left(1.007\text{∗}{\text{k}}_{\text{o}}\text{– }\left(\text{DSR∗}1.007\text{∗}{\text{k}}_{\text{d}}\right)\right)\right]\text{ – }\left[0.0246\text{∗N∗}1.05\left(1.007\text{∗}{\text{k}}_{\text{o}}\text{– }\left(\text{DSR∗}1.007\text{∗}{\text{k}}_{\text{d}}\right)\right)\right]\text{∗}22.26\text{,}$$

where N = N_o_ and DSR is defined in Equation 9 by the BM in kg.

For calculation purposes, this simplifies to

(Equation 11) $${\text{rCO}}_{2}=\left[0.45859\text{∗N∗}\left({\text{k}}_{\text{o}}\text{– }\left(\text{DSR∗}{\text{k}}_{\text{d}}\right)\right)\right]\text{∗}22.26.$$

The results of using this equation are shown in Table 2 (Equation 10), and a plot of the predicted rCO_2_ from Equation 10 and the observed rCO_2_ across all the validation studies across the entire weight range in Table 2 is shown in Figure 2B. This shows a linear relationship with an r^2^ of 90.1% and a least-squares fit gradient of 0.954 (reduced major axis = 1.005). The average % difference across all 34 individuals in the validation studies (in Table 2) using this equation was 0.64% (SD = 11.9). This combined equation based on the weight dependency of the DSR in the range 0–10 kg therefore performs better than the individual equations for the ranges 0–2 kg (Equation 6) and 2–10 kg (Equation 7; Table 2).

**Table 2:** Validation results for carbon dioxide production (rCO_2_) for 34 preterm and neonatal babies measured using the doubly labeled water method simultaneous to chamber calorimetry

Study	ID	BM	rCO_2_ IC	Equation 1		Equation 6		Equation 7		Coward 1985		Speakman 7.17		Equation 10	
		g	L/d	L/d	%diff	L/d	%diff	L/d	%diff	L/d	%diff	L/d	%diff	L/d	%diff
D	3	1,090.00	12.50	9.09	−27.27	11.4	−8.5	9.94	−20.49			12.80	2.40	12.85	2.81
B	3	1,115.00	11.37	10.00	−12.08	11.4	0.3	10.51	−7.63	8.53	−25.04	12.44	9.36	12.22	7.44
B	7	1,195.00	14.58	13.60	−6.74	15.3	4.8	14.20	−2.57	11.91	−18.32	16.60	13.85	16.16	10.82
D	2	1,378.00	13.70	13.64	−0.45	15.9	15.8	14.44	5.40			17.39	26.93	16.72	22.07
A	10	1,414.85	17.72	13.48	−23.91	15.8	−10.8	14.32	−19.18	14.59	−17.65	17.37	−1.98	16.65	−6.02
D	4	1,496.00	17.00	14.99	−11.85	17.8	4.7	16.00	−5.87			19.63	15.47	18.68	9.87
A	1	1,520.65	18.29	13.13	−28.24	15.4	−15.7	13.95	−23.71	12.90	−29.50	16.96	−7.29	16.10	−11.97
B	1	1,545.00	14.83	13.94	−6.03	16.2	9.2	14.75	−0.55	9.86	−33.50	17.74	19.63	16.82	13.40
A	5	1,596.45	19.74	19.16	−2.95	21.6	9.6	20.05	1.58	17.70	−10.36	23.53	19.22	22.25	12.73
B	4	1,600.00	15.52	14.61	−5.85	17.2	11.0	15.55	0.21	11.80	−24.00	18.94	22.07	17.86	15.08
B	6	1,640.00	18.70	17.48	−6.53	19.8	5.9	18.31	−2.04	15.12	−19.15	21.56	15.31	20.32	8.69
B	2	1,660.00	17.76	16.77	−5.58	19.5	9.7	17.75	−0.08	13.71	−22.79	21.35	20.21	20.05	12.88
A	8	1,692.15	20.01	18.01	−10.00	20.9	4.2	19.04	−4.87	18.38	−8.15	22.85	14.15	21.41	6.96
A	7	1,702.70	22.88	26.77	16.98	29.3	28.0	27.68	20.96	26.82	17.18	31.58	37.99	29.76	30.06
A	9	1,709.20	21.17	13.92	−34.24	16.5	−21.9	14.87	−29.79	15.35	−27.49	18.23	−13.89	17.01	−19.65
A	11	1,783.30	22.61	18.81	−16.79	21.6	−4.4	19.83	−12.31	19.35	−14.44	23.63	4.50	22.01	−2.64
A	12	1,824.10	21.17	18.87	−10.85	21.4	1.2	19.79	−6.50	19.78	−6.55	23.33	10.25	21.72	2.64
B	8	1,830.00	21.23	19.09	−10.09	21.8	2.6	20.06	−5.52	18.06	−14.93	23.75	11.89	22.09	4.04
B	5	1,860.00	18.97	15.54	−18.08	18.4	−3.2	16.56	−12.70	14.00	−26.19	20.22	6.60	18.65	−1.71
A	6	1,862.40	18.44	14.19	−23.06	16.8	−9.0	15.12	−17.99	14.76	−19.98	18.48	0.21	17.03	−7.63
A	4	1,880.70	25.36	22.30	−12.06	25.1	−0.9	23.32	−8.03	22.64	−10.74	27.32	7.74	25.39	0.10
A	3	1,894.95	25.47	12.37	−51.44	16.4	−35.7	13.81	−45.77	15.32	−39.84	18.53	−27.24	16.69	−34.47
C	6	1,920.00	21.95	21.99	0.16	25.7	17.3	23.34	6.33			28.27	28.77	25.99	18.40
A	2	1,996.80	23.04	19.40	−15.79	22.5	−2.5	20.50	−10.98	19.33	−16.09	24.60	6.81	22.56	−2.08
Mean		1,633.64	18.92	16.30	−13.45	18.9	0.5	17.24	−8.42	15.99	−18.38	20.71	10.12		
SD		252.89		4.07	13.54	4.30	13.40	4.15	13.45	4.37	11.97	4.60	14.07		
C	1	2,570.00	27.55	25.42	−7.75	30.7	11.4	27.32	−0.85			33.97	23.30	29.56	7.28
D	1	2,575.00	27.90	21.67	−22.33	25.7	−7.9	23.12	−17.14			28.31	1.45	24.82	−11.04
C	4	2,590.00	25.98	22.39	−13.83	25.7	−1.1	23.58	−9.24			28.08	8.05	24.97	−3.92
C	5	2,790.00	28.00	27.36	−2.28	31.9	13.8	28.98	3.50			34.93	24.75	30.55	9.11
C	8	2,980.00	32.70	27.02	−17.37	32.0	−2.3	28.80	−11.94			35.19	7.59	30.23	−7.55
C	9	3,390.00	33.82	33.14	−2.02	38.3	13.3	35.01	3.51			41.96	24.05	35.96	6.31
C	3	3,440.00	34.27	32.11	−6.32	36.9	7.6	33.83	−1.30			40.29	17.55	34.64	1.08
C	2	3,890.00	41.22	42.11	2.16	47.7	15.9	44.14	7.09			51.99	26.13	44.58	8.17
C	7	4,030.00	37.18	34.56	−7.04	41.5	11.7	37.08	−0.28			45.93	23.53	37.46	0.73
C	2b	4,160.00	50.40	46.29	−8.16	51.9	2.9	48.30	−4.17			56.27	11.65	48.48	−3.81
Mean		3,241.50	33.90	31.21	−8.49	36.2	6.5	33.02	−3.08			39.69	16.81	34.12	0.64
SD		627.09	8.49	8.69	12.00	8.8	8.1	9.08	11.99			10.67	12.82	8.63	12.17

The top half of the table refers to children weighing less than 2 kg (n = 24) and the bottom half those weighing more than 2 kg (n = 10). Study is the reference where the original validation data were published. A is Jensen et al.,^28^ B is Westerterp et al.,^27^ C is Jones et al.,^32^ and D is Roberts et al.^26^ ID is the ID from the original study. BM is the mean body mass of the individual in g. rCO_2_ IC is the indirect calorimetry estimate of CO_2_ production in liters per day. For each DLW equation, the original data were used to calculate rCO_2_ and the % difference between these estimates and the chamber CO_2_ production. At the bottom of each part of the table, the summary statistics across all individuals in each sub-group are shown. The summary statistics for Equation 10 refer to the whole sample of n = 34. Equations 1, 6, 7, and 10 refer to the equations derived in the text here. Coward 1985 refers to the two-pool equation in Coward and Prentice.^22^ Speakman 7.17 refers to Equation 7.17 in Speakman,^3^ which is the most widely adopted and validated equation for use in small mammals and birds. For some of the studies, N_d_ was not available from the original validations. Because the equation Coward 1985 requires individual estimates of N_d_, a comparison was not possible for these subjects.

Using the combination of Equations 9 and 10 (or 11) eliminates the boundary discontinuities of using three separate equations and provides a general equation for the estimation of rCO_2_ from DLW studies, the adult equation (Equation 1) being a special case of this more general solution where body mass is greater than 10 kg. A further benefit of this equation combination is that, if more refined analyses in the future result in equations that are better able to predict the DSR, these could be adopted by replacing Equation 9 with an updated prediction model.

We see considerable future benefits in studies using these new equations because they will improve the accuracy of the derived estimates of energy expenditure. Moreover, by having a single equation set that spans all body sizes, it will be easier for researchers to select the best calculation solution to get the most accurate outcomes. Finally, they will enormously facilitate the compilation and comparison of data across different studies. Indeed, we have already prepared a number of manuscripts based on these equations that consider diverse aspects of energy demands, including global aspects of nutrition, energy demands through the lifespan H.P. et al., unpublished data, impacts of physical activity on lean body mass and energy compensation strategies (V. Careau et al., unpublished data; K.R.W. et al., unpublished data, and trends in energy demands over time (J.R.S. et al., unpublished data To facilitate the adoption of these equations, we have also developed a dedicated website that is free to use where users can input isotope data to derive the rCO_2_ and TEE using the recommended procedures (http://dlw.som.cuanschutz.edu/).

We suggest that future studies using the DLW method should consider adopting a standard approach for calculating rCO_2_ and its conversion to TEE. For this purpose, we recommend in adults the equations adopted here (Equation 1 and its calculation forms in Equations 3 and 4) for calculating rCO_2_ and the Weir equation for the conversion of rCO_2_ to TEE (Equation 5). This recommendation is based on the performance of the rCO_2_ equation in adult validation studies (Table 1). In babies (<10 kg), we suggest adoption of Equation 10, where the dilution space ratio is calculated from body weight. This equation performs best in validation studies of babies. Alternatively, if these standards are not adopted, then we suggest users should make available in supplemental materials the values of k_o_, k_d_, N_o_, and N_d_ for each individual subject, so that the published estimates can be easily converted to the standard, thereby improving future comparisons. Moreover, we strongly advocate users to upload their DLW data into the IAEA DLW database^8^ and make their standardized data widely available to the scientific community.

### Limitations of study

The main advantage of the DLW method is that it allows a measure of free-living energy demands unencumbered by any measurement apparatus. The main advantage of the chamber indirect calorimetry approach is its verified precision and accuracy based on sound physiological and engineering principles. However, chamber calorimetry has the disadvantage that the range of activities that individuals can engage in is more limited than free-living subjects can perform. When the two techniques are brought together in a validation, it is expected because of the restricted activity that the energy expenditure of most subjects would sit at the low end of the spectrum of free-living demands, and hence, the validation may be biased to low levels of expenditure. However, the average CO_2_ production across all subjects in the validation study was 497.5 L/day (Table 1), which is comparable to the expected average CO_2_ production of adult free-living individuals weighing 80 kg in the IAEA database of 494 L/day. Hence, this is unlikely to be a serious source of bias. Perhaps the biggest weakness is the fact that, although on average, the new equations perform well at the individual level, there are still considerable discrepancies at the individual level. This variation limits utility of the method to measure individual levels of energy expenditure. The cause of this variation remains unclear and is generally presumed to reflect random errors in isotope enrichment determinations. However, the validation studies have generally not recorded the diets consumed by the subjects. Because, in theory, different dietary constituents may provide different opportunities for hydrogen isotope exchange and may stimulate different levels of *de novo* lipogenesis, this could contribute to isotope dilution spaces and fluxes that are not accounted for in the standard calculation, contributing to the individual discrepancies. Further validation work with individuals consuming known and quantified diets might contribute to lowering this error. As a final word of caution, there are no validation studies for individuals aged >70 years, and the dilution space ratio may decline at older ages.^20^ We suggest Equation 1 should be used in this age group with caution.

## Consortia

This consortia authorship contains the names of people whose data were contributed into the IAEA DLW database by the analysis laboratory, but they later could not be traced or they did not respond to emails to assent inclusion among the authorship. The list also includes some researchers who did not assent inclusion to the main authorship because they felt their contribution was not sufficient to merit authorship: Stefan Branth; Niels C. De Bruin; Lisa H. Colbert; Alice E. Dutman; Simon Eaton; Sölve Elmståhl; Mikael Fogelholm; Tamara Harris; Rik Heijligenberg; Hans U. Jorgensen; Christel L. Larsson; Margaret McCloskey; Gerwin A. Meijer; Daphne L. Pannemans; Renaat M. Philippaerts; John J. Reilly; Elisabet M. Rothenberg; Sabine Schulz; Amy Subar; Minna Tanskanen; Ricardo Uauy; Rita Van den Berg-Emons; Wim G. Van Gemert; Erica J. Velthuis-te Wierik; Wilhelmine W. Verboeket-van de Venne; and Jeanine A. Verbunt.

## STAR★Methods

### Key Resources Table

**Table:** 

REAGENT or RESOURCE	SOURCE	IDENTIFIER

**Deposited data**		

The data on which the analyses were based is available in the International Atomic Energy Agency Doubly labeled water database.	International Atomic Energy Agency	https://www.dlwdatabase.org/

**Software and algorithms**		

Software for calculating results of DLW experiments	University of Colorado	http://dlw.som.cuanschutz.edu/

### Resource availability

#### Lead contact

Further information and requests for resources and reagents should be directed to and will be fulfilled by the Lead Contact. John R Speakman (jspeakman@abdn.ac.uk)

#### Materials availability

This study did not generate new unique reagents.

#### Data and code availability

The data presented here pertain to the IAEA DLW database (v3.1) which is a repository of almost 7000 measurements of daily energy expenditure in humans made using the DLW method. Full details of the aims and scope of the database can be found in reference ^8^.

### Experimental model and subject details

The analysis here includes data for 5756 children, adolescents and adults and 1021 babies and infants extracted from the IAEA database v3.1. These data have all been published previously and are extracted from relevant publications for inclusion in the database by authors of those papers.

### Method details

This study is based on recalculation of previously published data concerning use of the DLW method in free-living subjects and in experiments involving DLW and simultaneous chamber indirect calorimetry. There is no standard approved protocol for the use of the DLW technique and hence studies vary in the exact methods employed. In general however subjects are dosed with ^18^Oxygen and deuterium in drinking water at a dose rate aiming to produce an excess enrichment of ^18^Oxygen between 150 and 300 ppm above background levels, and an enrichment of deuterium about half that. A background urine sample is taken prior to dosing and an equilibrium sample commonly 3-4 hours afterward (3^rd^ void) but in some protocols 10-12h later. The measurement duration can vary between 7 and 21 days and during that period samples may be collected only at the start and end, or on multiple occasions throughout the washout period. Measurement durations are generally shorter for children and dosing can be higher than for adults. The isotope washout is normally calculated from the log converted isotope enrichments above background. When multiple samples are collected it may also be evaluated from a non-linear exponential model fit to the data. Isotope dilution spaces may be calculated from the back extrapolated washout to the dose time, or from the equilibrium samples. During free-living studies individuals continue their daily routines as normal. Full details of the practical aspects of the method can be found in ref ^3^. During chamber validation studies the subjects live continuously or semi-continuously inside a room calorimeter. Semi-continuous occupancy is for 23.5h per day with 30 mins allowed outside for chamber calibration and for subjects to shower. Gas exchange from the chamber is measured using gas analysers and CO_2_ production calculated from the difference in CO_2_ content between incurrent and excurrent air and the flow rate.

### Quantification and statistical analysis

Measurements using different methods were compared in a pairwise fashion using the Bland-Altman methodology^26^. Comparisons between the simultaneous DLW and chamber respirometry values were made by calculating the absolute differences (precision) and summed differences including the sign (accuracy) between DLW estimates of CO_2_ production derived from different equations and the chamber indirect calorimetry estimates.

## References

[1] Lifson N., Gordon G.B., McCLINTOCK R. (1955). Measurement of total carbon dioxide production by means of D2O18. J. Appl. Physiol..

[2] Lifson N. (1966). Theory of use of the turnover rates of body water for measuring energy and material balance. J. Theor. Biol..

[3] Speakman J.R. (1997). Doubly Labelled Water: Theory and Practice.

[4] Nagy K.A. (1983). The Doubly Labeled Water (3HH18O) Method: A Guide to Its Use.

[5] Schoeller D.A., van Santen E. (1982). Measurement of energy expenditure in humans by doubly labeled water method. J. Appl. Physiol..

[6] Westerterp K.R., Saris W.H.M., van Es M., ten Hoor F. (1986). Use of the doubly labeled water technique in humans during heavy sustained exercise. J Appl Physiol (1985).

[7] Klein P.D., James W.P., Wong W.W., Irving C.S., Murgatroyd P.R., Cabrera M., Dallosso H.M., Klein E.R., Nichols B.L. (1984). Calorimetric validation of the doubly-labelled water method for determination of energy expenditure in man. Hum. Nutr. Clin. Nutr..

[8] Speakman J.R., Pontzer H., Rood J., Sagayama H., Schoeller D.A., Westerterp K.R., Wong W.W., Yamada Y., Loechl C., Murphy-Alford A.J. (2019). The International Atomic Energy Agency International Doubly Labelled Water Database: aims, scope and procedures. Ann. Nutr. Metab..

[9] Dugas L.R., Harders R., Merrill S., Ebersole K., Shoham D.A., Rush E.C., Assah F.K., Forrester T., Durazo-Arvizu R.A., Luke A. (2011). Energy expenditure in adults living in developing compared with industrialized countries: a meta-analysis of doubly labeled water studies. Am. J. Clin. Nutr..

[10] Schoeller D.A., Allison D.B., Schoeller D.A., Westerterp-Plantenga M.S. (2017). Advances in the Assessment of Dietary Intake.

[11] International Atomic Energy Agency (2009). IAEA Human Health Series No. 3. Assessment of Body Composition and Total Energy Expenditure in Humans Using Stable Isotope Techniques.

[12] Schoeller D.A., Webb P. (1984). Five-day comparison of the doubly labeled water method with respiratory gas exchange. Am. J. Clin. Nutr..

[13] Westerterp K.R., Brouns F., Saris W.H., ten Hoor F. (1988). Comparison of doubly labeled water with respirometry at low- and high-activity levels. J. Appl. Physiol..

[14] Seale J.L., Conway J.M., Canary J.J. (1993). Seven-day validation of doubly labeled water method using indirect room calorimetry. J Appl Physiol (1985).

[15] Schoeller D.A., Ravussin E., Schutz Y., Acheson K.J., Baertschi P., Jéquier E. (1986). Energy expenditure by doubly labeled water: validation in humans and proposed calculation. Am. J. Physiol..

[16] Ravussin E., Harper I.T., Rising R., Bogardus C. (1991). Energy expenditure by doubly labeled water: validation in lean and obese subjects. Am. J. Physiol..

[17] Melanson E.L., Swibas T., Kohrt W.M., Catenacci V.A., Creasy S.A., Plasqui G., Wouters L., Speakman J.R., Berman E.S.F. (2018). Validation of the doubly labeled water method using off-axis integrated cavity output spectroscopy and isotope ratio mass spectrometry. Am. J. Physiol. Endocrinol. Metab..

[18] Schoeller D.A. (1988). Measurement of energy expenditure in free-living humans by using doubly labeled water. J. Nutr..

[19] Racette S.B., Schoeller D.A., Luke A.H., Shay K., Hnilicka J., Kushner R.F. (1994). Relative dilution spaces of 2H- and 18O-labeled water in humans. Am. J. Physiol..

[20] Sagayama H., Yamada Y., Racine N.M., Shriver T.C., Schoeller D.A., DLW Study Group (2016). Dilution space ratio of 2H and 18O of doubly labeled water method in humans. J Appl Physiol (1985).

[21] Speakman J.R., Nair K.S., Goran M.I. (1993). Revised equations for calculating CO2 production from doubly labeled water in humans. Am. J. Physiol..

[22] Coward W.A., Prentice A.M. (1985). Isotope method for the measurement of carbon dioxide production rate in man. Am. J. Clin. Nutr..

[23] Shanthini R. (2006). Working with ideal gas. Thermodynamics for Beginners with Worked Examples.

[24] Weir J.B. (1949). New methods for calculating metabolic rate with special reference to protein metabolism. J. Physiol..

[25] Bland J.M., Altman D.G. (1986). Statistical methods for assessing agreement between two methods of clinical measurement. Lancet.

[26] Wells J.C., Ritz P., Davies P.S., Coward W.A. (1998). Factors affecting the 2H to 18O dilution space ratio in infants. Pediatr. Res..

[27] Roberts S.B., Coward W.A., Schlingenseipen K.H., Nohria V., Lucas A. (1986). Comparison of the doubly labeled water (2H2(18)O) method with indirect calorimetry and a nutrient-balance study for simultaneous determination of energy expenditure, water intake, and metabolizable energy intake in preterm infants. Am. J. Clin. Nutr..

[28] Westerterp K.R., Lafeber H.N., Sulkers E.J., Sauer P.J.J. (1991). Comparison of short term indirect calorimetry and doubly labeled water method for the assessment of energy expenditure in preterm infants. Biol. Neonate.

[29] Jensen C.L., Butte N.F., Wong W.W., Moon J.K. (1992). Determining energy expenditure in preterm infants: comparison of 2H(2)18O method and indirect calorimetry. Am. J. Physiol..

[30] Ritz P., Johnson P.G., Coward W.A. (1994). Measurements of ^2^H and ^18^O in body water: analytical considerations and physiological implications. Br. J. Nutr..

[31] de Bruin N.C., Degenhart H.J., Gàl S., Westerterp K.R., Stijnen T., Visser H.K. (1998). Energy utilization and growth in breast-fed and formula-fed infants measured prospectively during the first year of life. Am. J. Clin. Nutr..

[32] Jones P.J.H., Winthrop A.L., Schoeller D.A., Swyer P.R., Smith J., Filler R.M., Heim T. (1987). Validation of doubly labeled water for assessing energy expenditure in infants. Pediatr. Res..

[33] Speakman J.R. (1993). How should we calculate CO_2_ production in doubly labeled water studies of animals?. Funct. Ecol..

